# Dentition and Its Derangements. Lecture X, Part 2

**Published:** 1862-10

**Authors:** A. Jacobi


Dentition and its Derangements.—*By A. Jacobi*, M.D. —*LECTURE X*—Part 2.—The anatomical structure of both the normally growing and the rhachitical bone is the same; further, the amount of carbonic acid is increased in both the new deposits of the growing and in the rhachitical bone. Thus the direct connection between, or dependency on each other, of the normal development and the rhachitical deformity of the osseous tissue is evident.

The principal deviation of the chemical constituents consists in the increased amount of water. Friedleben found in the healthy parietal bone of a child of six months 21·058 per cent. of water; in the rhachitical parietal bone at the same age, from 48·383 to 53·574 per cent.; and in that of a rhachitical child of eight months, as much as 81·533 per cent. The occipital bone of a child of a year and two months, with normal skeleton, yielded 41·931 per cent. of water, while the parietal bone of a rhachitical child of the same age yielded 66·314 per cent. in the recent deposits, the old subjacent lamina vitrea yielding but 34·221 per cent. The same author found in the normal diaphysis of the tibia of a healthy child of six months, 21·323 per cent. of water; of a rhachitical one, at eight months, 44·790; in the spongeous part of the former, 64.037; of the latter, 76·912. The same bones of children of a year and two months, one healthy, the other rhachitical, yielded a similar proportion, viz.: 43 and 72 per cent. The normal ribs of a child of six months contained 44.305 per cent. of water, the rhachitical, 54·809. In other children this percentage would increase to 59·64, and even to 66·105.

The increased amount of water contained in the rhachitical bone forms a sort of serous mollification, which in itself may be sufficient to prevent the cartilage and newly formed bone from assimilating as many phosphates as under normal circumstances. For it appears, doubtless, that with so considerable an amount of water contained in the osseous and cartilaginous tissues, their chemical affinity generally should be changed. At all events, an older explanation of the rhachitical bone being deprived of a part of its phosphates is very improbable. Many authors have expressed this opinion, that the phosphates were kept dissolved in the blood by a superabundance of lactic acid, and were, in company with the latter, eliminated through the kidneys and bladder. A part of the chemical investigations which have been made for the purpose of elucidating this matter, have appeared to be favorable to this opinion, as not only lactic acid, but also a large amount of phosphate of lime, has been found in the urine of many rhachitical children. But in order to explain the want of phosphates in rhachitical bone, in this manner, the presence of phosphates and the occurrence of lactic acid in the urine of rhachitical children ought to be constant; which is by no means so in all cases. Rhachitis will very often develop without the prevalence of disorders of digestion and a surplus of lactic acid, and surely, the swelling of the epiphysis and periosteum, which are just as essential for the diagnosis of rhachitis, can not be explained by the premature elimination of phosphate of lime. Virchow, moreover, has long ago expressed this opinion, that probably the diminished import of phosphate was of more importance in the rhachitical process than the augmented export. At the same time he directs the attention to the repeated eulogies of the administration of carbonate and phosphate of lime, in rhachitis. He also reminds us of the fact, that the larger part of phosphates are introduced into the system in combination with proteinous substances, and the digestion and assimilation of these latter are greatly interfered with in those gastric disorders, which frequently precede fully developed rhachitis. But again, he asks, why it is that gastric disorders are not always the initiatory step of rhachitis; that further, the bones should suffer in preference; and finally, that, in spite of diminished import, the epiphyses and periosteum should be tumefied. Therefore, we need not wonder that other authors, for instance Niemeyer, consider as the fundamental cause of rhachitis, a nutritive disorder in the epiphyses and periosteum kindred to inflammation. This author points to the fact, that in other tissue, as skin and mucous membrane, we frequently meet with diffuse affections of exanthematous or catarrhal nature, the causes of which are totally unknown to us, but which, though not constantly, are principally found in cachetic, badly nourished, and rhachitical children. And that, after tumefactions have once commenced, the impeded circulation should prevent the phosphates from being deposited to a sufficient amount, and favor their immediate elimination through the kidneys; that therefore the usual superabundance of phosphates in the urine of rhachitical children must be taken as consequence rather than as cause of the want of phosphates in the diseased tissue, is but natural; especially after those remarks I have made before, on the influence of the increased amount of water in the osseous and cartilaginous tissue.

To what extent Virchow is right in directing our attention to the connection between rhachitis, which is very common among the poorer class of society, and the absence of proteinous substances from the food, either breast-milk or artificial, is shown by everybody's and every day's experience. Böcker found in the milk of a mother, who nursed a rhachitical child until it died at the age of fifty-three days, in 1000 parts, but 13·111 of casseine, 23·31 of butter, 60·358 of sugar, and only traces of phosphates to the amount of 0·089. Friedleben has, in his "Contributions to the knowledge of the physical and chemical contributions of growing and rhachitical bones, in early infancy," the following remarks:— I examined the milk of two women, whose children were brought up with breast milk exclusively. The skeletons of both were rhachitical in their totality; both recovered, but only after their diet had been changed. One was a turgid, pale woman, of forty-six years, who had been bled several times during her pregnancy. Two specimens of her milk were examined, the first in the fifth, the other in the sixth month after the birth of her child. The analysis yielded, in 100 parts, in

| No. 1. | No. 2. |
|---|---|
| Water, 87·829. | Water, 87·830. |
| Butter, 4·390. | Butter, 4.390. |
| Caseine and sugar, 7·542. | Caseine and sugar, 7·542. |
| Inorganic matter, 0·239. | Phosphate of lime, 0·069. |
| | Alkaline salts, 0·169. |

The milk of the other woman, four months and a half after the birth of her child, looked thin and serous like that above. Since her confinement, being at work and without medical attendance, she had been suffering from uterine hemorrhages. She was tall and slender, and nearly forty years old. The analysis of her milk resulted in the following figures. In 100 parts of milk there were,

| | |
|---|---|
| Water | 91·307 |
| Caseine, butter, sugar | 8·509 |
| Phosphate of lime | 0·099 |
| Carbonate of lime | 0·010 |
| Alkalies | 0·073 |
| Oxide of iron and silicium | 0·002 |

The normal proportions in the healthy milk, not to speak of constitutional and other oscillations, differ considerably from the above, as is proved by the following figures of the several percentages of normal milk:

| | |
|---|---|
| Water | 86·60 |
| Caseine | 3·50 |
| Sugar | 6·20 |
| Butter | 3·50 |
| Soluble Salts | 0·06 |
| Insoluble Salts | 0·14 |

From these statements it follows, that the milk of both women was below the average rate of proteine and hydrates of carbon. The earthy salts were also diminished, but they were fewer in the first case than in the second, while the rha chitis of the second was more decidedly developed than that of the first. Nothing else is required to prove, that the diminished import of phosphates is neither the only nor the principal cause of rhachitis.

At all events then, this much is understood now, that there are other important elements in what we have been used to call rhachitis besides the diminished amount of phosphates ; and that want of phosphates, and rhachitis, are by no means identical. Nor is the very nature of rhachitis explained by, or comprehended in, the process of resorption. This is best shown by those physiological experiments which allow a free absorption, but less access of earthy salts and phosphoric acid. Chosat has made the first series of such experiments. Friedleben made similar ones, allowing a number of pigeons no other food but vetches and distilled water. Through five or six months they appeared to be well; then diarrhea would set in, emaciation take place, and death from exhaustion ensue in the tenth month. The chemical analysis of their bones, compared with those of healthy pigeons, resulted in the following figures :—

| Pigeon. | Bone. | Inorganic matter. | Organic matter. | Fat. | Carbonic Acid. | Spec. gravity when dried at 360°. | Spec. gravity when dried in open air. |
|---|---|---|---|---|---|---|---|
| Healthy ........ | diaph. humor | 64.603 | 35.397 | 7·110 | 8·571 | 2·692 | 1·822 |
| Diseased......... | diaph. ulnæ, et radii. | 37.712 | 62.288 | 12·084 | 6·842 | 2·038 | 1.762 |

A further difference was this, that jelly could be formed of the bones of the healthy pigeon in eleven, of the diseased in five minutes. Thus, as gluten was the organic basis in either, the chemical constitution of the diseased bones, so far as organic matter was concerned, was not at all altered. But there were a number of other differences between the diseased bone and the rhachitical tissue. The amount of earthy salts decreased to nearly one-half of the normal percentage ; fat increased; specific gravity less. Carbonic acid twenty-five per cent. less than in the bones of the healthy bird. No tumefaction of epiphyses or periosteum. These results depend on diminished assimilation, which, moreover, is distinctly proved by anatomical examination of the diseased bones.

They are thin, fragile, not flexible; they are anæmic, and their medullary canals very wide indeed; the surface is uneven; there are intervals, interruptions, between the remnants of the bone and the dispersed osseous corpuscles, which are much less numerous and less regular than normal, to such a degree that Haversia canals between them are not recognizable. There was nowhere a trace of recent formation, only the proofs of normal absorption.

Now, after having sifted to some extent whatever is known to this very moment on the anatomical and chemical nature of rhachitis, the most recent results included, and alluded to the usual alleged causes of this disease, I need hardly return to my former assertion, that dentition as such, and rhachitis, are in no causal relation to each other; at all events there is no such connection between the two that the process of the protrusion of teeth could produce rhachitis. Not to speak again of the want of phosphates alone not constituting rhachitis, the amount of these salts slowly assimilated for the formation of some teeth is very small in proportion to what is contained in the food. But surely, the largely diminished amount of phosphates introduced into the system (not only into the stomach), as being the result of the rhachitical process, well explains the slowness with which the teeth form and protrude in rhachitical children, and the simultaneous retardation of walking, and the slow ossification of bones generally. At another place, and in a former lecture also, I have spoken at some length of these matters. Thus we need not wonder that a rhachitical child who has no tooth at a year or two, can not walk at the normal period, nor that its cranial sutures and fontanelles are not closed before the second or third year. But these latter anomalies do not depend on the retardation of the appearance of teeth, but all of them—retardation of the closure of the cranial bones, retardation of walking, and retardation of the protrusion of teeth—all of them are to be considered as the cotemporaneous and co-ordinate results of the same fundamental morbid process.—*Am. Medical Times.*

